# Updates on the Association Between Vitiligo and Thyroid Diseases: A Systematic Review

**DOI:** 10.7759/cureus.69697

**Published:** 2024-09-19

**Authors:** Riyad M Abuhalimeh, Layan Saeed Alshmrani, Nourah Abdullah, Assem Mubarak H Alqahtani, Sereen Dhafer AlQarni, Mohannad Muaid Aljuaid, Waleed Khalid Z Alghuyaythat, Saud Sheher Mohammed Alkahtani, Afnan F Aljawi, Ghena Mahir Abdulrahman Alsaadi

**Affiliations:** 1 Department of Dermatology, Saudi Ministry of Health, Arar, SAU; 2 College of Medicine, King Khalid University, Abha, SAU; 3 College of Medicine, University of Bisha, Bisha, SAU; 4 Department of Medicine, Prince Sattam bin Abdulaziz University, Al Kharj, SAU; 5 Department of Medicine, King Khalid University, Abha, SAU; 6 Department of Otorhinolaryngology, National Guard Hospital, Khobar, SAU; 7 College of Medicine, Majmaah University, Al-Majmaah, SAU; 8 Department of Emergency Medicine, Riyadh First Health Cluster, Riyadh, SAU; 9 Department of Medicine and Surgery, Taibah University, Medina, SAU; 10 College of Medicine, King Abdulaziz University, Jeddah, SAU

**Keywords:** autoimmune comorbidity, depigmentation, systematic review, thyroid disorders, vitiligo

## Abstract

The main objective of this study is to estimate the prevalence of thyroid diseases in patients with vitiligo and investigate the potential shared autoimmune mechanisms underlying the co-occurrence of vitiligo and thyroid diseases. To locate research that met the inclusion criteria, a thorough computerized search of relevant databases was carried out. A comprehensive search was carried out on PubMed, SCOPUS, Science Direct, and Web of Science to locate relevant material. Our data included 13 trials with 82,230 participants, and 40,116 (48.8%) of them were males. The prevalence of thyroid disorders ranged from 3.2% to 32.1%, with a total prevalence of 2,906 (3.5%). Vitiligo patients are more likely to have a number of immunological comorbidities, underscoring the serious effects of the illness on overall health, especially thyroid disorders. The correlation between vitiligo and positive thyroid peroxidase antibodies, hypothyroidism, and autoimmune thyroiditis is notably high. We found a strong association between vitiligo and the incidence of thyroid disorders, particularly autoimmune thyroid disorders. The findings emphasize the necessity of identifying and treating thyroid dysfunction in vitiligo patients, as it might affect the clinical course of the skin condition and overall patient health. Future research is required to standardize study methodology, investigate underlying mechanisms, and create integrated therapy and screening regimens.

## Introduction and background

About 1% of people worldwide suffer from vitiligo, a common, acquired cutaneous condition that causes depigmentation [1,2]. There is no preference for sex or ethnicity; the condition affects both adults and children. Onset typically occurs in the second or third decade of life [3].

The precise etiology of vitiligo is still unknown. The autoimmune theory is the most accepted explanation, supported by a number of epidemiological, clinical, and experimental evidence [4-6]. These findings suggest that melanocyte abnormalities initiate the pathophysiology of vitiligo by inducing an autoimmune reaction that results in the loss of melanocytes in susceptible people. Compared to the general population, vitiligo patients are more likely to experience autoimmune diseases [7].

There has been a suggestion of a connection between vitiligo and autoimmune thyroid disorders (AITDs) [8]. Compared to healthy people, vitiligo patients have been shown to have a higher prevalence of thyroid problems, both clinical and subclinical [9,10]. AITD is characterized by raised levels of antibodies towards antigens specific to the thyroid, such as thyroid peroxidase (TPO) and thyroglobulin (Tg).

Research has demonstrated that the prevalence of thyroid disorders was higher in vitiligo patients compared to the general population [9]. This finding could point to a shared autoimmune cause. For those who have vitiligo, early detection and treatment of thyroid abnormalities are essential as they may exacerbate the disease or negatively impact overall health. Although a connection has been established, research has shown differing degrees of correlation, making it difficult to determine the exact nature of the relationship [9,10]. To identify research gaps, synthesize existing knowledge, and give a thorough understanding of the association between thyroid disorders and vitiligo, a systematic evaluation of the literature is required. This study aims to determine the prevalence of thyroid diseases in vitiligo patients.

## Review

Methods

We implemented this systematic review in line with the Preferred Reporting Items for Systematic Reviews and Meta-Analyses (PRISMA) [11] criteria. An internet-based search was performed on PubMed, Web of Science, SCOPUS, and Science Direct to find English-language studies on the association between thyroid disease and vitiligo. The search technique in these cases made use of pertinent keywords. Separate reviewers went through the search results, selected pertinent papers, gathered information, and applied the proper evaluation techniques to determine the quality of the included study. By independently extracting important information and critically evaluating the included research's quality using established assessment procedures, these reviewers made sure that reliable studies and data were selected for further evaluation and summary in this systematic review.

Eligibility criteria are represented in Table 1.

**Table 1:** Eligibility criteria for included studies

Criterion	Inclusion	Exclusion
Focus	Studies reporting the prevalence of thyroid disorders among vitiligo patients; studies discussing the underlying mechanisms	Studies not focusing on the association between thyroid disorders and vitiligo
Study Design	Research printed in peer-reviewed publications; recent studies (2014-2024); studies conducted on human subjects	Animal or in vitro studies; reviews, case reports, editorials, and opinion pieces
Language	Studies available in the English language	Studies not available in the English language

Data Extraction

Rayyan (Qatar Computing Research Institute, Doha, Qatar) was utilized to check the search results and ensure accuracy [12]. The inclusion and exclusion criteria were employed to establish the relevance of the titles and abstracts in the search results. The study team thoroughly reviewed papers that met the inclusion criteria. Consensus was employed to resolve disputes. Key study data were recorded using an established data extraction form, including study titles, authors, year of publication, study setting, participant demographics, prevalence of thyroid disorders, and primary outcomes. To investigate the probability of bias, a neutral evaluation instrument was developed.

Strategy for Data Synthesis

Data from relevant studies were used to produce descriptions of the research findings and features, which allowed for a qualitative review. After the data acquisition for the systematic review was completed, the optimal plan to ensure the utilization of the data from the included studies was determined.

Risk of Bias Assessment

The Joanna Briggs Institute (JBI) [13] critical assessment criteria designed for studies reporting prevalence data will be applied to evaluate the quality of the research included in this investigation. Nine questions make up this tool, and the answers are ranked as either positive (rated as one) or negative (scored as zero), unclear, or irrelevant. Based on total ratings that fall below four, between five and seven, and above eight, studies were categorized as poor, moderate, or high quality, respectively. Researchers independently assessed the quality of the studies they undertook, and any disagreements in the evaluations were settled by cooperative discussion to guarantee agreement and precision in the quality assessment procedure.

Results

Systematic Search Outcomes

**Figure 1:**
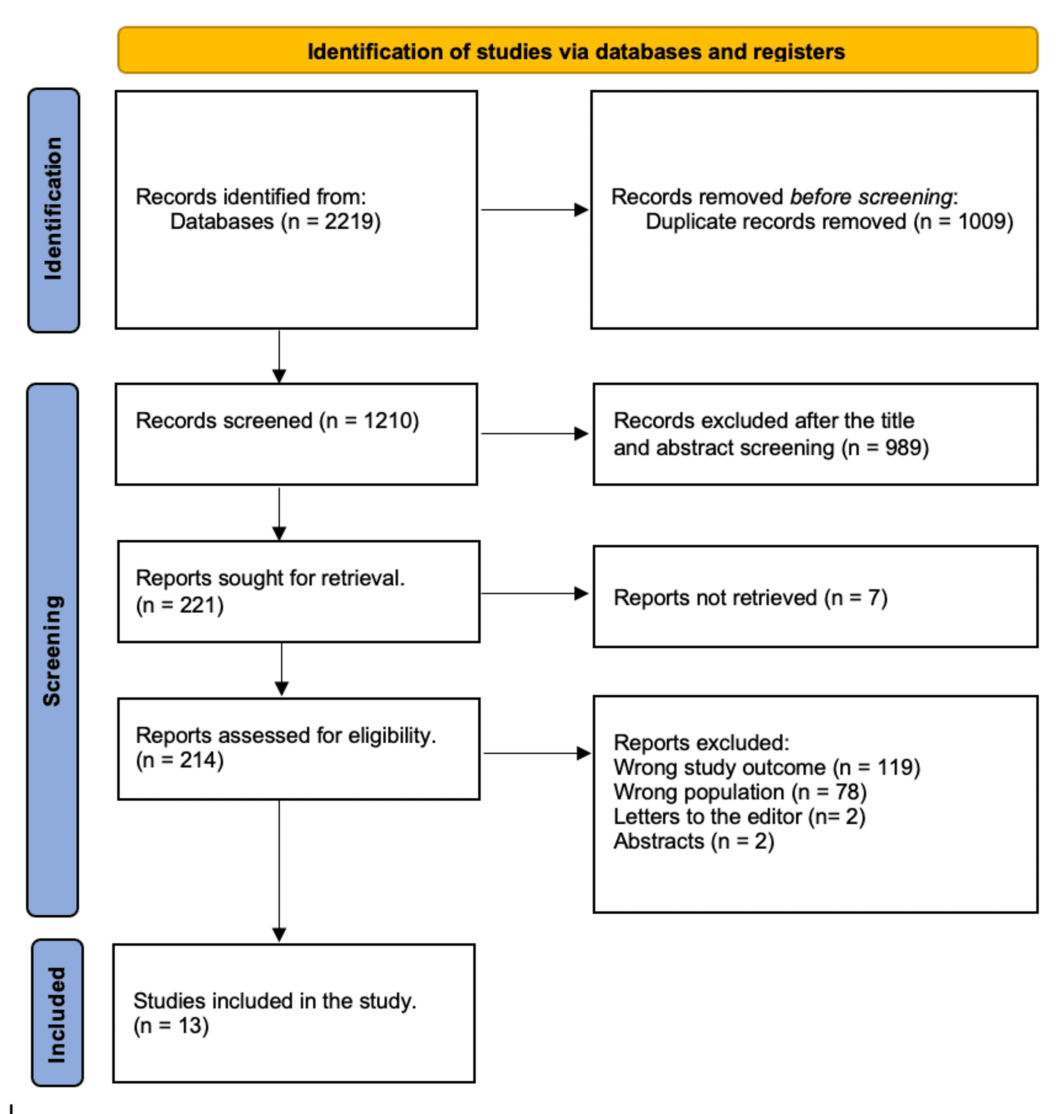
A Preferred Reporting Items for Systematic Reviews and Meta-Analyses (PRISMA) diagram is used to summarize the study decisions.

Following the removal of 1,009 duplicates, a systematic search yielded 2,219 study papers. After 1,210 studies' titles and abstracts were reviewed, 989 papers were rejected. Out of the 221 reports that needed to be obtained, seven articles were not found. Two hundred fourteen articles passed the full-text screening procedure; 119 were dismissed as the study results were improper, 78 because the type of population was incorrect, two were editor's letters, and two were abstracts. The eligibility requirements were satisfied by 13 research publications that have been incorporated in this systematic review. Figure 1 depicts a diagram of the approach used to select the literature. 

Sociodemographic of the Comprised Participants and Studies

Table 2 displays the sociodemographic information from the research articles. Our data included 13 trials with 82,230 participants, and 40,116 (48.8%) of them were males [14-26]. Four studies were retrospective cohorts [14-15,22,25], four were cross-sectional studies [16-17,20,23], three were case-control studies [18,22,24], one was a retrospective observational study [26], and one was a retrospective cohort [19]. Four studies were conducted in India [18-19,24,26], one in Israel [14], one in Spain [15], one in Malaysia [16], one in Pakistan [17], one in Korea [20], one in Thailand [21], one in Saudi Arabia [22], one in the USA [23], and one in Belgium [25].

**Table 2:** Sociodemographic parameters of the involved populations NM, not mentioned

Study	Study design	Country	Participants	Mean age	Males (%)
Ramot et al., 2024 [14]	Retrospective cohort	Israel	11,412	42.3 ± 21.3	5,579 (48.9)
Estebaranz et al., 2024 [15]	Retrospective cohort	Spain	1,400	40.7 ± 19.7	645 (46.1)
Teh et al., 2024 [16]	Cross-sectional	Malaysia	62	35.5 ± 44	23 (37.1%)
Ashraf et al., 2021 [17]	Cross-sectional	Pakistan	125	31.6 ± 7.8	41 (56.8%)
Agarwala and Malkud, 2020 [18]	Case-control	India	261	25.95 ± 1.08	128 (49%)
Latha and Saheb, 2019 [19]	Prospective cohort	India	120	34.41 ± 13	45 (35%)
Bae et al., 2017 [20]	Cross-sectional	Korea	73,336	NM	32,519 (44.3%)
Vachiramon et al., 2017 [21]	Retrospective cohort	Thailand	325	NM	119 (36.6%)
Al Houssien et al., 2017 [22]	Case-control	Saudi Arabia	122	45 ± 19	34 (27.9%)
Gill et al., 2016 [23]	Cross-sectional	USA	1,098	45.5	508 (46.3%)
Gopal et al., 2014 [24]	Case-control	India	150	24 ± 10.28	83 (55.3%)
van Geel et al., 2014 [25]	Retrospective cohort	Belgium	699	32.7	333 (47.6%)
Saha et al., 2024 [26]	Prospective observational study	India	120	28.8 ± 17.9	59 (49.2%)

Clinical Outcomes

The clinical data are presented in Table 3. The prevalence of thyroid disorders ranged from 3.2% [16] to 32.1% [17], with a total prevalence of 2,906 (3.5%). Vitiligo patients are more likely to have a number of immunological comorbidities, underscoring the serious effects of the illness on overall health, especially thyroid disorders. The correlation between vitiligo and positive TPO antibodies, hypothyroidism, and autoimmune thyroiditis is notably high.

**Table 3:** Clinical parameters and outcomes of the comprised research JBI, Joanna Briggs Institute

Study ID	Intervention	Main outcomes	JBI
Ramot et al., 2024 [14]	488 (4.3%)	Vitiligo patients are likely to have a number of immunological and psychological associations, underscoring the serious effects of the illness on overall health.	Moderate
Estebaranz et al., 2024 [15]	127 (9.1%)	This study validates the link between high illness burden and comorbidities (such as eczema and thyroid issues) and vitiligo.	Moderate
Teh et al., 2024 [16]	2 (3.22%)	Reduced moisture in vitiligous skin points to a compromised epidermal barrier. Biophysical traits or itching were not linked to thyroid antibody positivity.	Moderate
Ashraf et al., 2021 [17]	41 (32.1%)	In patients with generalized vitiligo, the incidence of positive thyroid peroxidase antibodies is considerable.	High
Agarwala and Malkud, 2020 [18]	32 (12.3%)	The correlation between hypothyroidism and vitiligo is notably high.	Moderate
Latha and Saheb, 2019 [19]	18 (16.7%)	Patients with vitiligo are more likely than the general population to have autoimmune thyroid disorders, most often hypothyroidism and autoimmune thyroiditis that are validated by the presence of antithyroid antibodies.	Moderate
Bae et al., 2017 [20]	1822 (2.5%)	Male patients and younger patients typically showed stronger relationships. This is the first population-based study that identifies overt autoimmune thyroid disorders in real life using pertinent medications.	Moderate
Vachiramon et al., 2017 [21]	102 (31.4%)	The results of other worldwide studies are in line with the high incidence of AITD and positive thyroid antibodies in vitiligo patients. It is essential to screen for AITD in vitiligo patients by utilizing thyroid antibodies and blood TSH levels.	High
Al Houssien et al., 2017 [22]	32 (26%)	In comparison with the control group, vitiligo patients showed a significantly greater frequency of hypothyroidism.	Moderate
Gill et al., 2016 [23]	135 (12.3%)	They discovered that the prevalence of thyroid disease was higher (12.9%, p< 0.001)	Moderate
Gopal et al., 2014 [24]	30 (20%)	Vitiligo, autoimmune hypothyroidism, and diabetes mellitus are clearly related.	Moderate
van Geel et al., 2014 [25]	67 (9.6%)	The only autoimmune/autoinflammatory disease that has the potential to be used as a clinical criterion to somewhat predict the clinical development of vitiligo is thyroid disease.	Moderate
Saha et al., 2024 [26]	10 (8.3%)	The illness is linked to cutaneous disorders and other autoimmune diseases.	High

Ramot et al. (2024) found that vitiligo patients frequently experience both psychological and immunological associations, reflecting the disease's profound effects on overall health [14]. Additionally, the work by Estebaranz et al. (2024) validates a strong connection between the burden of vitiligo and comorbidities like eczema and thyroid issues, underlining the complexity of managing this condition [15].

Several studies delve into the prevalence of AITDs among vitiligo patients. Ashraf et al. (2021) reported a significant incidence of TPO antibodies in those with generalized vitiligo, indicating a robust relationship between these conditions [17]. Similarly, Latha and Saheb (2019) noted higher occurrences of AITDs, particularly hypothyroidism and autoimmune thyroiditis, in individuals with vitiligo [19]. The correlation with hypothyroidism was further echoed by Agarwala and Malkud (2020), who documented a notably high correlation [18].

Moreover, Bae et al. (2017) presented population-based evidence linking AITDs to vitiligo, particularly emphasizing the outcomes observed in younger male patients [20]. Vachiramon et al. (2017) aligned with earlier studies, advocating for routine screening of autoimmune thyroid disease in vitiligo patients through thyroid antibodies and thyroid-stimulating hormone (TSH) blood levels [21]. Lastly, other systemic connections between vitiligo and autoimmune diseases were confirmed through the study by Saha et al. (2024), integrating a broader perspective on the condition's impact [26].

Discussion

The prevalence of thyroid disorders ranged from 3.2% [16] to 32.1% [18], with a total prevalence of 3,145 (3.5%). This was lower than Fan et al., who reported a 15.7% prevalence of thyroid disease and a 1.9% prevalence of AITD [27]. Vitiligo patients are more likely to have a number of immunological comorbidities, underscoring the serious effects of the illness on overall health, especially thyroid disorders. The correlation between vitiligo and positive TPO antibodies, hypothyroidism, and autoimmune thyroiditis is notably high. We propose that it is important to take thyroid antibody levels in otherwise seemingly healthy people seriously. Because anti-thyroid antibodies are quite prevalent in vitiligo patients, routine blood tests should be carried out, and patients with positive results should be monitored on a regular basis.

Our review made clear that vitiligo's clinical course could be made worse by thyroid problems. For example, hyperthyroidism may initiate or exacerbate the depigmentation process, whereas hypothyroidism may affect the skin's capacity to repair and respond to treatments. Given the reciprocal nature of these illnesses, it is likely that optimal treatment outcomes for vitiligo patients depend on controlling thyroid function in addition to general health. Vrijman et al. found a raised prevalence and risk of AITD in vitiligo patients compared to non-vitiligo patients. It seems that this risk increases with age. Physicians should be aware that patients with vitiligo have an increased risk of thyroid illness and should watch for signs [28]. Fan et al. stated that physicians should be aware that vitiligo patients are at a higher risk for AITD [27]. Similarly, Yuan et al. also found that subclinical hyperthyroidism and Graves’ disease had the lowest prevalence, whereas subclinical hypothyroidism had the greatest. Monitoring vitiligo patients for thyroid disorders is feasible in an effort to detect prospective thyroid diseases or evaluate the risk of future development [29].

This study has a few drawbacks. The pooled studies used various inclusion/exclusion criteria, sampling methods, definitions of vitiligo and thyroid disease, and, most crucially, different areas and races. As a result, there may have been differences among the research participants in terms of iodine intake, genetic background, age, and clinical judgment by attending physicians. 

Future studies should include longitudinal investigations to determine the temporal link between the emergence of vitiligo and thyroid illness. This would assist in determining whether one condition predisposes people to develop the other or if they occur separately as part of a larger autoimmune process. Further investigation of the genetic and immunological mechanisms that link vitiligo and thyroid illness is required. Studies on specific genetic variants, immune cell profiles, and autoantibody synthesis could shed light on the disorders' similar etiology.

## Conclusions

This comprehensive review found a strong link between vitiligo and thyroid problems, particularly AITDs. The findings emphasize the necessity of identifying and treating thyroid dysfunction in vitiligo patients, as it might affect the clinical course of the skin condition and overall patient health. Future research should attempt to standardize study methodology, investigate underlying mechanisms, and create integrated therapy and screening regimens.
